# Evaluation of accuracy and precision of CT-guidance in Radiofrequency Ablation for osteoid osteoma in 86 patients

**DOI:** 10.1371/journal.pone.0169171

**Published:** 2017-04-06

**Authors:** H. Nijland, J. G. Gerbers, S. K. Bulstra, J. Overbosch, M. Stevens, P. C. Jutte

**Affiliations:** 1 Department of Orthopaedics, University of Groningen, University Medical Center Groningen, Groningen, Netherlands; 2 Department of Radiology, University of Groningen, University Medical Center Groningen, Groningen, Netherlands; George Washington University, UNITED STATES

## Abstract

**Background and purpose:**

Osteoid osteoma is a benign skeletal tumour that accounts for 2–3% of all bone tumours. The male-to-female ratio is around 4:1 and it predominates in children and young adults. The most common symptom is pain, frequently at night-time. Historically the main form of treatment has been surgical excision. With the development of Radiofrequency Ablation (RFA) there is a percutaneus alternative. Success rates of RFA are lower but the main advantage is the minimal invasive character of the therapy and the low complication rate. As a result of the minimal invasiveness the hospitalization- and rehabilitation periods are relatively short. However, in current literature no values for accuracy and precision are known for the CT-guided positioning.

**Methods:**

Accuracy and precision of the needle position are determined for 86 procedures. Furthermore the population is divided into groups based on tumour diameter, location and procedure outcome.

**Results:**

The clinical success rate was 81.4%. In 79% of procedures complete ablation was achieved. Accuracy was 2.84 mm on average, precision was 2.94 mm. Accuracy was significantly lower in more profound lesions. Accuracy in tibia and fibula was significantly higher compared to the femur. No significant difference was found between different tumour diameters.

**Interpretation:**

The accuracy and precision found are considered good. Needle position is of major importance for procedure outcomes. The question however rises how the results of this therapy will turn out in treatment of larger tumours.

## Introduction

Osteoid osteoma is a benign skeletal tumour that accounts for 2–3% of all bone tumours[1] and 10–12% of all benign bone tumours [2,3]. This tumour has low growth potential and usually a diameter less than 15mm [2]. Osteoid osteoma can develop on multiple locations, but are most frequently seen in the long bones of the lower extremity, i.e. femur, tibia and fibula [1]. Osteoid osteoma is characterized by a radiolucent nidus surrounded by a variable degree of sclerosis. The male-to-female ratio is around 4:1 and it predominates in children and young adults [3]. The most common symptom is pain localized in the bone which is most frequent during night-time. This pain can be relieved by using non-steroidal anti-inflammatory drugs (NSAID’s) [1].

The main form of treatment of these tumours has, for a long time, been classical surgery. Hereby the options are curettage, en bloc resection and wide resection (with grafting). The success rate of classical surgery ranges from 88–100% [4]. However, the main point of concern is the occurrence of complications like avascular necrosis of the femoral head and fractures [3,4]. According to Cantwell et al. complications occur in 20–45% of procedures. Besides these complications mean surgery time is longer and tissue damage, scarring and morbidity are higher compared to minimally invasive therapies [4]

Minimal invasive treatment for osteoid osteoma can be done with Radiofrequency Ablation (RFA). RFA as a treatment for osteoid osteoma was first described in 1992 [5].In RFA therapy a tumour is thermally ablated by a needle-shaped electrode. This needle is placed through a drilled tract based on CT-images (i.e. CT-guidance) [1]. The position of the needle is essential for optimal destruction without complications. When the tumour is within 15mm of a neurovascular structure classical surgery is the treatment of first-choice [4]. The main advantage of RFA compared to other forms of treatment is the minimal invasive character of the therapy in combination with the corresponding low complication rate [6]. As a result of the minimal invasive character the hospitalization- and rehabilitation periods are relatively short [7].

Success rates for RFA, defined as being free of symptoms 2 weeks after treatment, range from 76–100%[4]. Although this success rate is lower compared to classical surgery (88–100%), RFA is currently considered to be the main treatment option mainly due to the low invasiveness of the treatment [8]. It is likely that the success rate of RFA can be improved by (more) accurate positioning of the needle(accuracy defined as the minimal distance between the needle and the nidus of the tumour). Complication rates can potentially be reduced by (more) precise positioning (precision defined as the closeness of agreement of this distance between different patients) [8]. In current literature no values for accuracy and precision of CT guided RFA needle placements are known, although they are likely to be a major factor in success rates. Therefore the main purpose of this paper is to determine and evaluate the accuracy and precision of freehand positioning under CT-guidance, the current standard technique for RFA needle guidance. The accuracy and precision are also determined for subgroups based on diameter and location of the tumour. Besides this, the typical use of CT-guidance is analysed by means of secondary parameters (i.e. placement time, angulation, tumour depth, number of RFA tempi, number of runs, number of residues, complications, and the clinical success rate).

## Methods

**Fig 1:**
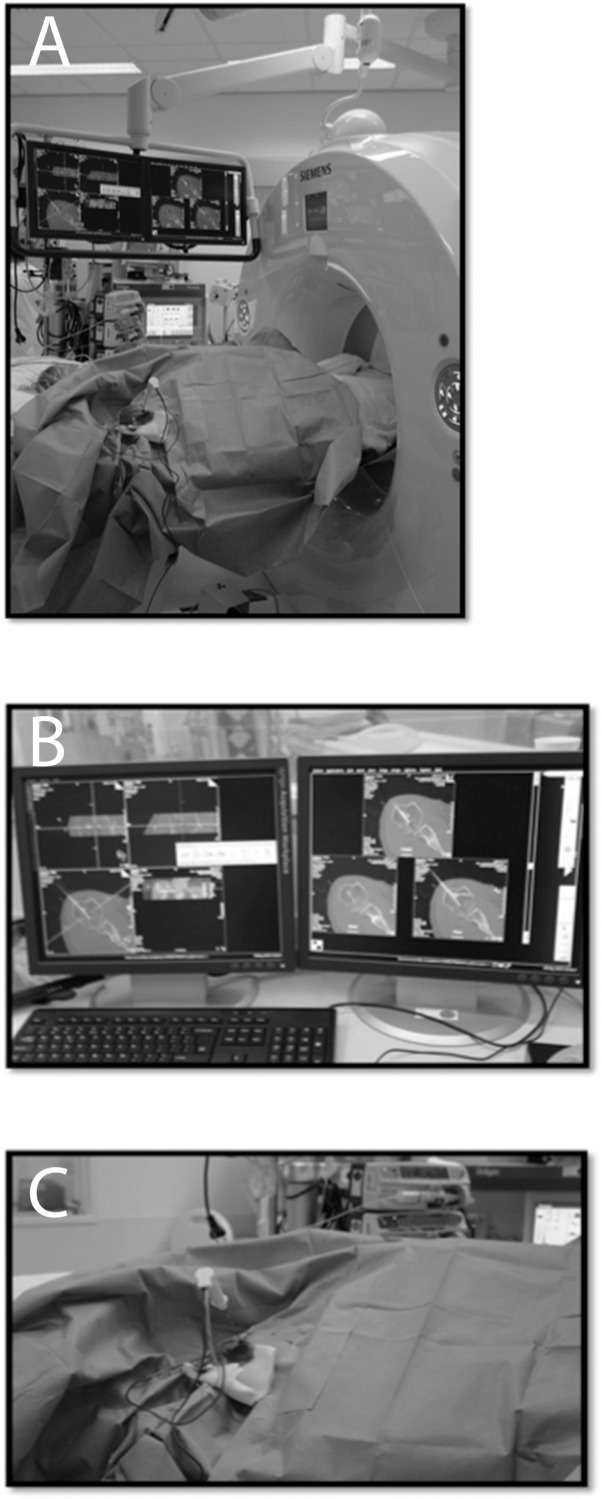
**RFA procedure** A: Set-up.B: Planning needle position. C: Needle in situ during ablation.

**Fig 2:**
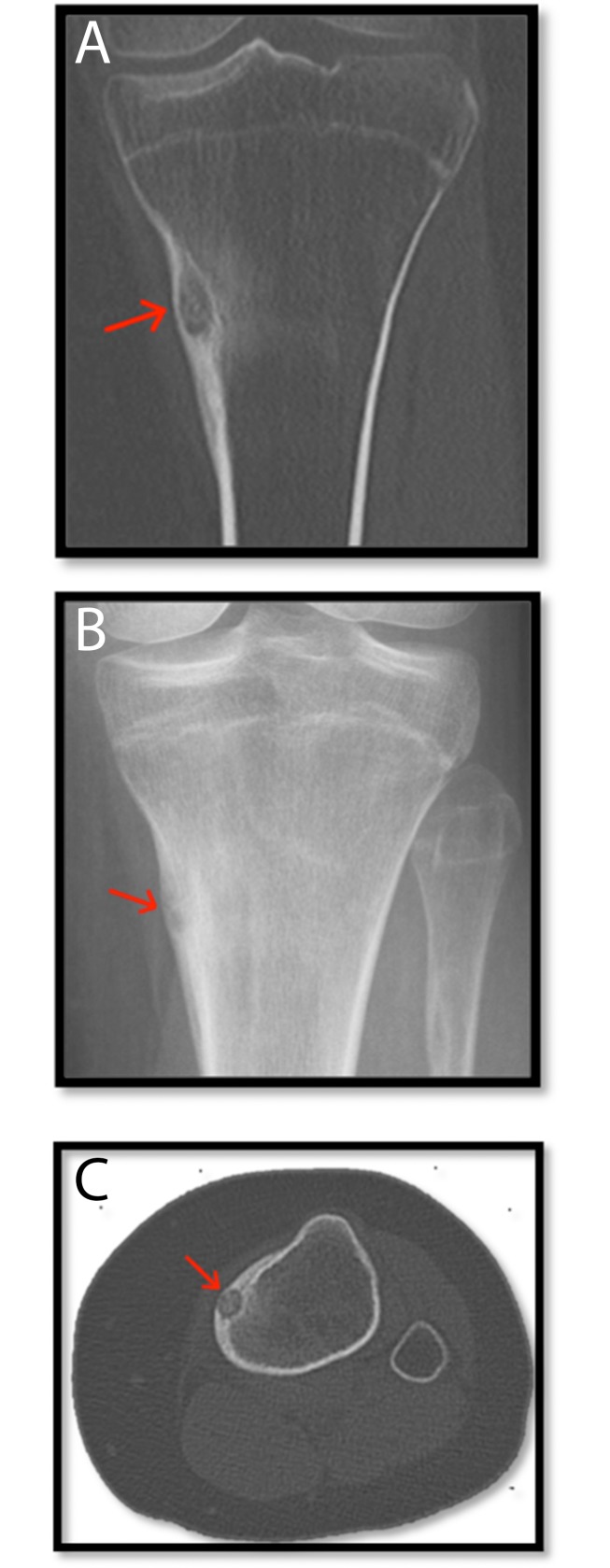
A-C. Osteoid osteoma in Tibia.

**Fig 3:**
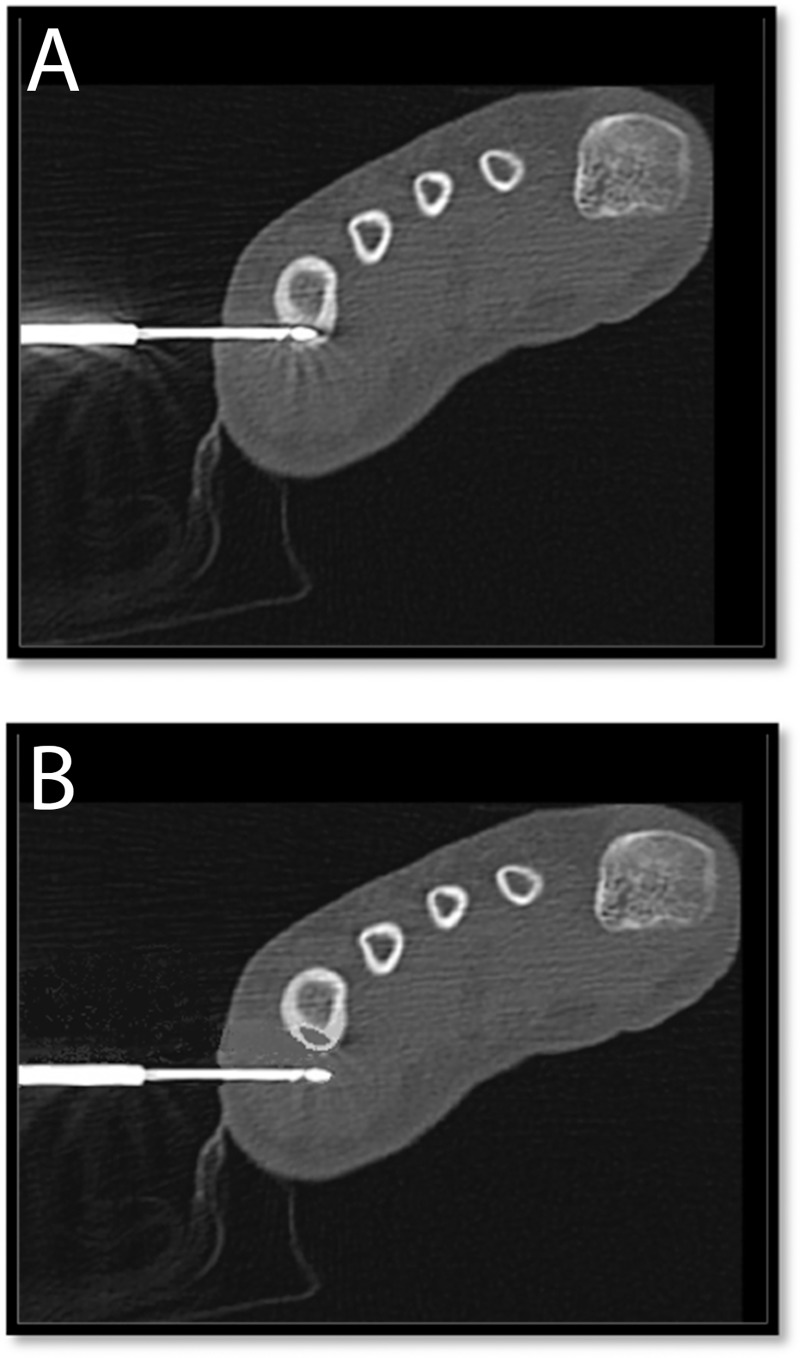
CT images of an ablation needle in situ. **Osteoid osteoma in 5th metatarsal.** A: Needle in nidus (accurate placement). B: Needle outside nidus (= inaccurate placement).

All data is obtained from a prospectively kept bone tumour database of University Medical Center Groningen (UMCG) on the criteria of having had a RF-ablation for osteoid osteoma. Data of patients treated with RFA in the last 10 months is analysed with Orthomap 3D software (Stryker, MahWah, United States) and added to the database. In accordance to regulations of the Medical Ethical Review Board of University Medical Center Groningen, patients were informed by means of written information about the fact that anonymous data of the procedure could be used for scientific research. As the procedure was part of usual care no written or verbal consent was necessary. If patients had objections to the use of their data the names of these patients were documented and consequently these data were not included in the study. Figs 1–3 depict an osteoid osteoma and the RFA set-up and procedure.

### Measurements

**Fig 4:**
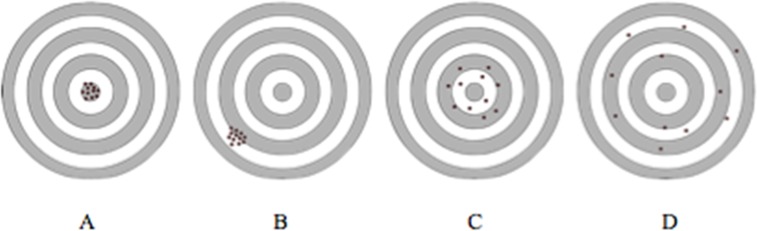
Formulation of accuracy and precision. A: high accuracy, high precision, B: high accuracy, low precision, C: low accuracy, high precision, D: low accuracy, low precision.

In this study accuracy and precision are defined in accordance with the ISO 5725–1 norm [9]. Accuracy is formulated as the minimal distance between the needle and the nidus of the tumour and expressed in mm. The precision is formulated as the closeness of agreement of this distance between different patients and is measured as the standard deviation of the accuracy and expressed in mm. These measures are depicted in Fig 4.

In order to obtain more information about the accuracy and precision on different tumour sizes, the population has been separated into 2 groups based on the diameter of the tumour. Group 1 comprised tumours with a diameter of ≤10 mm and group 2 contained tumours of >10 mm. The purpose of this separation is to evaluate the effect of tumour size on ablation effect rate. To compare the course and effects of the RFA procedure between different locations, patients are divided into 3 separated groups (femur, tibia and fibula).

After identifying the accuracy and precision for the entire population as well as for subgroups based on diameter and location, we evaluated whether there is a difference in accuracy, precision and procedure outcomes between the subgroups. By means of possible variances in these outcomes we look at the importance of accurate positioning.

Secondary parameters are placement time, angulation, tumour depth, number of RFA tempi, number of runs, number of residues, complications, and the clinical success rate. The placement time is the period between the first and last run. The angulation is formulated as the angle between the true path of the needle and the shortest possible route from the chosen entry point to the nidus. RFA tempi is defined as the number of ablations during a procedure and the clinical success rate is defined as being free of pain 2 weeks after the procedure.

### Statistical analysis

All data is analysed with SPSS Statistics v20 (IBM, *Armonk*, *United States)*. For the comparison between the groups based on diameter a Mann-Whitney U test is used. For locations a Kruskal-Wallis test is used. Fisher’s exact test is used for the variables on an ordinal scale (i.e. residue, complications, clinical success) for the diameter groups. For these variables in the location groups a Chi-square test is used. For tests on precision Levene’s test is used. Pearson’s test is used to determine correlations. For all tests an alpha of 0.05 was chosen.To control alpha for multiple group comparisons (for locations) it is divided by the number of comparisons, in this case by 3[10].

## Results

### Demographics

Table 1 displays the demographics of the patient population and the basic data about the tumours (i.e. diameter, depth, side, location). A number of 59 from 86 patients were male (2.2:1 ratio). The mean age of patients was 26.1(±10.7) years. In 5 of the 86 analysed procedures, a complication occurred. In 2 cases this comprised a skin burn, in 2 cases an infection occurred and finally 1 patient had a fracture.

**Table 1:** Demographics & basic tumour data.

**Number of procedures**	86
**Sex (m/v)**	59/27
**Follow-up (months)**	54.1 (±30.6)
**Age (years)**	26.1 (±10.7)
**Diameter nidus (mm)**	8.6 (±4.5)
**Depth (mm)**	43.9 (±24.3)
**Side (left/right)**	40/46
**Location (femur/tibia/fibula/other)**	31/29/9/17*

Mean values (± deviation).

* = number of cases per group.

### Accuracy & precision

Table 2 shows the accuracy, precision and angulation for the total group and for group 1 (≤10mm) compared to group 2 (>10mm). The accuracy of the needle position appeared to be 2.84 mm on average for all patients together. The precision was found to be 2.94 mm. There was a mean angulation of 4.9°(±3.31) compared to the shortest route from entry point to nidus. Nine Patients (10.5%) had a residue. From the 86 included patients 70 no longer reported pain 2 weeks after the procedure. Therefore the clinical success rate was 81.4%.

**Table 2:** Population separated in groups based on tumour size.

		*Diameter group*		
	Total (N = 86)	≤10 mm (N = 62)	>10 mm (N = 24)	P-value
**Distance needle to tumour (mm)**	2.84 (±2.94)	2.5 (±2.8)	3.7 (±3.3)	0.161
**Placement time (min)**	36.0 (±17.4)	35.5 (±17.8)	37.3 (±16.9)	0.350
**Angulation (degrees)**	4.9 (±3.31)	4.8 (±3.5)	5.1 (±2.8	0.266
**Depth (mm from entry)**	43.9 (±24.3)	40.2 (±22.8)	54.0 (±25.9)	0.035
**Number of RFA tempi**	2.35 (±0.68)	2.35 (±0.75)	2.35 (±0.49)	0.612
**Number of runs**	7.85 (±2.93)	8.1 (±3.0)	7.3 (±2.8)	0.176
**Number of residues**	9	8	1	0.223
**Number of complications**	5	3	2	0.537
Free of pain** (y/n)	70/16	49/13	21/3	0.283

Mean values (± deviation).

** 2 weeks after procedure.

α = 0.05.

Values for accuracy, precision were smaller for group 1, but the outcomes show no significant differences with P values of 0.161 for accuracy and 0.266 for angulation. From the evaluated secondary parameters only the depth turned out to be significantly different between the groups (P = 0.035) with the larger tumours situated more profound. The number of RFA tempi and runs did not differ between the different tumour sizes. In the small diameter group the number of residues was distinctly higher, but with regard to group sizes no significant difference was found (P = 0.223).

In Table 3 the population is separated based on tumour location. First, the distance between needle and tumour (i.e. the accuracy) differs between the locations (P = 0.018) although non-significant (α = 0.017 after correction). Second the depth of tumours in the femur (almost) doubles the average depth of tumours in tibia and fibula (P < 0.001). The correlation between depth and needle-tumour distance is 0.395.

**Table 3:** Population separated in groups on location.

	*Location*			
	Femur (N = 31)	Tibia (N = 29)	Fibula (N = 9)	P-value
**Distance needle to tumour (mm)**	3.9 (±3.5)	1.8 (±2.1)	2.9 (±1.7)	0.018
**Placement time (min)**	37.8 (±17.6)	32.7 (±14.2)	33.1 (±14.1)	0.439
**Diameter (mm)**	10.1 (±5.0)	7.5 (±4.8)	7.6 (±2.6)	0.014
**Angulation (degrees)**	3.8 (±2.3)	5.4 (±3.3)	6.1 (±4.5)	0.098
**Depth (mm from entry)**	61.7 (±25.6)	29.9 (±10.8)	39.1 (±18.7)	<0.001
**Number of RFA tempi**	2.45 (±0.57)	2.14 (±0.71)	2.67 (±0.71)	0.119
**Number of runs**	7.6 (±3.0)	7.2 (±2.0)	7.8 (±2.9)	0.920
**Number of residues**	2	4	2	0.317
**Number of complications**	2	1	2	0.165
**Free of pain (y/n)**	24/7	22/7	8/1	0.702

Mean values (± deviation).

α = 0.017.

Furthermore the average diameter of tumours in the femur is significantly larger compared to the diameters on the other locations (P = 0.014). Finally no significant differences can be found in precision, placement time, angulation, the number of RFA tempi, runs, residues, complications and the clinical success rates.

**Fig 5:**
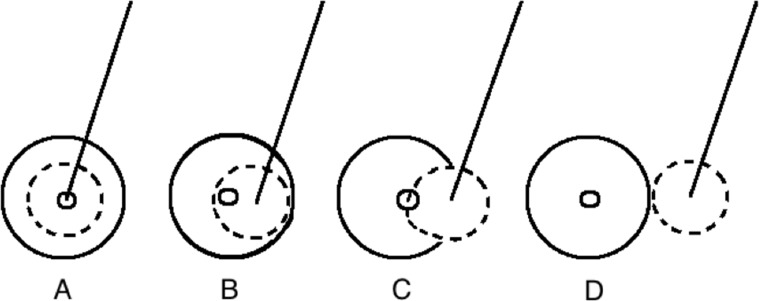
The ablation success categories. A: In nidus, complete. B: In centre, complete. C: In centre, incomplete. D: Not in centre, incomplete.

In Fig 5 the position of the needle and the halo around it are depicted for 4 groups dependent on the outcomes of the procedure. These groups are the ablation success categories. In Table 4 it can be seen that 68 of 86 procedures resulted in complete ablation (79.1%) of the tumour with 10 of these procedures with the needle placed within the nidus of the tumour (11.6%). The diameter of the nidus in lesions with incomplete ablation is significantly larger than the diameter of the nidus in lesions with complete ablation (P < 0.001). The Pearson correlation between diameter and ablation success categories is 0.63. Finally, even though it looks like tumours with incomplete ablation are located deeper under the skin, the depth of the tumour is not significantly different between the 4 groups (P 0.053).

**Table 4:** Population separated in groups on procedure accuracy and outcome.

	*Ablation success categories*				
	Group 1 (N = 10)	Group 2 (N = 57)	Group 3 (N = 15)	Group 4 (N = 4)	P-value
**Distance needle-tumour (mm)**	0,0 (±0,0)	2,3 (±2,0)	5,6 (±3,6)	8,3 (±4,0)	<0.001
**Diameter nidus (mm)**	7,2 (±3,3)	7,1 (±2,6)	12,3 (±4,4)	22,7 (±2,50	<0.001
**Placement time (min)**	30,0 (±12,7)	36,2 (±18,0)	39,7 (±19,0)	33,7 (±11,5)	0.481
**Angulation (degrees)**	3,3 (±2,0)	4,8 (±3,4)	6,1 (±3,6)	6,2 (±1,7)	0.066
**Depth (mm from entry)**	32,4 (±15,9)	41,0 (±22,8)	55,1 (±24,8)	80,9 (±30,8)	0.053
**Number of RFA tempi**	2,5 (±0,5)	2,3 (±0,8)	2,4 (±0,50)	2,0 (±0,0)	0.575
**Number of runs**	7,3 (±1,8)	8,2 (±3,2)	7,0 (±2,6)	6,7 (±0,6)	0.341
**Number of residues**	1	7	0	1	0.414
**Number of complications**	0	3	1	1	0.345
**Free of pain (y/n)**	7/3	46/11	15/0	2/2	.075

Group 1: Needle in nidus, complete ablation.

Group 2: Needle in centre, complete ablation.

Group 3: Needle in centre, incomplete ablation.

Group 4: Needle not in centre, incomplete ablation.

Mean values (± deviation).

α = 0.0125.

## Discussion

The goal of the current study was to identify and evaluate the accuracy and precision of needle position in freehand CT-guided radiofrequency ablation for the treatment of osteoid osteoma. The accuracy and precision of CT-guidance in RFA needle placement were found to be 2.84 mm and 2.94 mm respectively. The expected treatment radius is 5mm (ablation halo = 10 mm). Since this value exceeds the accuracy we found, complete tumour ablation can be expected for most patients. However, when considering the accuracy together with tumour diameter the treatment radius is no longer enough for large tumours, resulting in incomplete ablation.

Clinical success rate was in accordance with literature[4]. A number of 70 patients from the 86 included (81.4%) no longer reported pain 2 weeks after the procedure. The major advantage of RFA compared to classical surgery is the low number of complications and residues. In the current study we faced 5 complications in 86 procedures (5.8%) and 10.5% of the patients had a residue. In classical surgery complications occur in 20–45% of procedures [4]. Furthermore the complications in this study were of low impact, for example the infections comprised cellulitis and the fracture was a small fibula fracture with no long-term immobilisation.

### Depth and diameter

Factors that can potentially influence procedural accuracy, such as lesion depth and diameter have been investigated. With regard to the low correlation of 0.395 between depth and needle-tumour distance it cannot be stated that accuracy is lower as a result of tumour depth. Furthermore this data is not due to an increase in the number of runs to reach more profound lesions. As can be seen in Table 2 there is no significant difference in the accuracy of needle position between tumours with a small and large diameter (P = 0.161). This finding is in accordance with the differences in diameter between the different ablation success categories in Table 4. As can be seen, the diameter is significantly larger in tumours where the result of the procedure was incomplete ablation (P < 0.001). Most likely this is the result of the limited size of the ablation halo, which is around 10mm in cortical bone [11]. More data is necessary to examine this decrease. The precision of needle placement was not significantly better. It is interesting that operators did not use more RFA tempi for larger lesions.

### Location

Location of the lesion is another potential factor in procedural accuracy. From the data the number of RFA tempi in the fibula was higher compared to the other locations, although not significant (P = 0.119). Since there is no difference in tumour size between lesions in the fibula compared to the other locations or in distance of the needle to the tumour, the explanation for this might be fear of damaging the peroneal nerve. The use of smaller ablation zones may also be the explanation of the relatively high incidence of residues.

### Pain and needle position

In the data of Table 4 it is notable that the number of patients reporting pain 2 weeks after the procedure is higher in the category ‘needle in nidus’ compared to the categories with the needle into the centre (but not in the nidus). For procedures with the needle placed outside the centre of the tumour the percentage of patients reporting pain 2 weeks after the procedure is higher compared to the other groups. With regard to the small amount of procedures with the needle outside the centre this difference is not significant (P = 0.075). This observation however indicates it is necessary to position the needle within the centre of the tumour (high accuracy, high precision) to obtain elimination of the pain (clinical outcome). Since pain is the main problem in osteoid osteoma this position should be the hallmark of treatment.

### Larger lesions require multiple ablations

The importance of accurate positioning is even more emphasized in the treatment of larger tumours, with diameters exceeding the treatment radius around the needle (e.g. atypical cartilaginous tumours). In the treatment of these tumours multiple ablation points are necessary [11]. To ensure complete overlap between the ablation halos it is essential to accurately position the needles. The remaining question is whether CT-guidance is accurate and precise enough for multiple point ablations. The data provided in this study can be a basis for a CT-guided multi-point ablation treatment plan.

Alternative techniques can be the use of (virtual) fluoroscopy, Computer Assisted Surgery (CAS) or 3D printed guides. Not many studies have been published in this field [4]. An experimental study on 5 patients using (live guidance) fluoroscopy overlaying on CT data achieved a median accuracy of 0.6 mm [7].

### Conclusion

This is the first study to investigate the positional accuracy and precision of CT-guidance in RFA procedures in a large patient population with long follow-up. Data can be used to plan and perform safer (multiple point) ablations by compensating for known inaccuracy. Future research should focus on more accurate positioning (e.g. pre-planning, image fusion, and guidance), especially to enable multiple ablation points.

## Supporting information

S1 File DatasetAnonEnglish.sav dataset. (SAV)
